# Cognitive load, affect, and regulatory strategies: a more integrated model

**DOI:** 10.3389/fpsyg.2026.1774619

**Published:** 2026-05-28

**Authors:** Rebecca B. Brockbank, David F. Feldon, Kaylee Litson

**Affiliations:** 1 Department of Instructional Technology & Learning Sciences, Utah State University, Logan, UT, United States; 2 Department of Psychology, University of Houston, Houston, TX, United States

**Keywords:** cognitive reappraisal, affect, affective cognitive load, cognitive load, emotion regulation, mixed methods

## Abstract

Cognitive load theory (CLT) has become a powerful framework for conceptualizing learning from instruction. An emerging body of CLT literature has begun the examination of relationships between cognition and affective elements in recent years, broadening its scope. This exploratory study examines relationships between the cognitive load experienced in learning contexts, emotional states, motivational constructs, and regulatory strategies. Using a convergent parallel design, both quantitative and qualitative associations between affective constructs, including use of emotion regulation strategies, and differences in cognitive load and learning were explored within a descriptive longitudinal study. Findings suggest maladaptive affect may impose a measurable extraneous load component, and adaptive regulation may reduce it, offering preliminary empirical support for an affective extension of CLT. Further, study results reveal emotion regulation may not be an “add-on” to cognitive load management, but a fundamental mechanism through which participants navigated demanding learning tasks. Of the regulatory strategies examined, cognitive reappraisal emerged as a powerful strategy when load and emotional costs were highest. Findings point toward a more integrated framework for understanding learning that encompasses cognition, affect, motivation, and self-regulation as mutually constitutive processes, including the proposal of affective cognitive load (ACL) as a theoretically motivated subtype of extraneous cognitive load (ECL). To the best of the authors’ knowledge, this is among the first studies to combine CLT with affective dimensions of learning within a mixed methods, repeated measures framework.

## 1 Introduction

Grounded in an understanding of human cognitive architecture, cognitive load theory (CLT; Sweller et al., 1998, 2019) is foundationally supported by decades of research surrounding working memory (Baddeley, 1992; Cowan, 2001; Miyake and Shah, 1999), mental effort (Salomon, 1984), and schema construction (McVee et al., 2005; Paas et al., 2004). Yet for all its contributions to understanding of the cognitive elements of learning, its engagement with the interacting role of affect is still an emerging trend. This study examines the integral relationships between cognitive load and affect and the potential benefits of utilizing adaptive regulatory strategies such as cognitive reappraisal (Brockbank and Feldon, 2024).

The cognition/affect gap in CLT literature exists despite discussion and evidence of the ways in which affect may influence cognitive load and cognitive load may influence affect, including motivational constructs (Brockbank and Feldon, 2024; Feldon et al., 2019, 2024; Plass and Kalyuga, 2019; Plass and Kaplan, 2016). Further, CLT research has historically focused primarily on cross-sectional, quantitative inquiry. To expand the scope of the theory and its implications for practice, this study examines the relationships between the cognitive load experienced in learning contexts and emotional states and motivational constructs, exploring interactions between load and psychosocial factors impacting well-being, including affect, motivational constructs, and regulatory strategies. As a longitudinal mixed methods study, it examines these relationships as multi-directional over time.

We address the following research questions:

1. Does cognitive load mediate the relationship between affect and learning?
2. Does affect mediate the relationship between cognitive load and learning?
3. Does the use of adaptive regulatory strategies at the relationship between cognitive load and affect in learning? If so, which strategies facilitate desired outcomes?

## 2 Theoretical framing

### 2.1 Cognitive load theory foundations

Originally introduced in the 1980s, cognitive load theory (CLT) asserts that learning can be defined as the alteration of long-term memory through schema construction and automation (Kirschner et al., 2006). Further, working memory – a limited-capacity system supporting the cognitive processes of storage processing, rehearsal, maintenance, and executive control – is critical to a wide range of cognitive and social processes (Yang et al., 2013). Essentially, the premise of CLT asserts that because working memory is limited in both duration and capacity, only small amounts of novel, interacting elements can be effectively processed or encoded in long-term memory at any given time (Sweller, 2011; Sweller et al., 2019). Consequently, when instruction “overloads” working memory through either duration or capacity, schema construction is impaired. To facilitate effective learning, instruction must minimize such cognitive overload.

Cognitive load theory distinguishes between intrinsic cognitive load (ICL; i.e., inherent task demand) and extraneous cognitive load (ECL; i.e., unnecessary instructional burden) (Kalyuga, 2011; Sweller, 2010). Because ICL and ECL are additive, load that exceeds working memory capacity impairs learning (van Merriënboer and Sweller, 2005). Critically, whereas ICL is mitigated by learner expertise (e.g., prior knowledge), ECL can be directly reduced through improved instructional and environmental design. Importantly, both ECL and ICL can lead to cognitive overload, and it is possible they are related in differential and nuanced ways (Sweller et al., 2019).

### 2.2 Extending CLT: motivation, affect, and regulation

Recent CLT research has integrated motivation through expectancy-value-cost theory (Barron and Hulleman, 2015; Feldon et al., 2018, 2019, 2024; Seufert et al., 2024; see also Eccles and Wigfield, 2023). Expectancy (i.e., ability beliefs) and task values (i.e., attainment, intrinsic, utility) shape learning experiences, as do negatively perceived motivational costs associated with given learning tasks, particularly emotional/psychological costs, defined as negative affective states (e.g., stress, anxiety, overwhelm) arising during effortful tasks.

Despite these advances, CLT has largely treated cognition and affect as separate processes (see Brockbank and Feldon, 2024; Sweller et al., 2019), even though cognitive science establishes that emotions are integral to learning, influencing attention, memory encoding, reasoning, judgment, problem-solving, and cognitive flexibility (Ben-Eliyahu, 2019; Brockbank and Feldon, 2024; Hawthorne et al., 2025; Pekrun and Linnenbrink-Garcia, 2022). A critical gap remains regarding how learners’ affective states shape their subjective experience of cognitive load during learning, and whether adaptive emotion regulation strategies can mitigate ECL.

Further, recent research has integrated tenets of self-regulation and regulatory strategies within CLT (de Bruin et al., 2020, de Bruin and van Merriënboer, 2017; Hawthorne et al., 2019, 2025). Research suggests learners may increase regulatory strategy use in response to rising task difficulty, with growing information availability and associated cognitive demands posing particular challenges as learners simultaneously manage mental effort and self-regulation (de Bruin and van Merriënboer, 2017; de Bruin et al., 2020).

## 3 Situating affect and emotion regulation within CLT

Affect encompasses emotions and moods, along with associated physiological responses, that shape cognitive, motivational, and behavioral states in learning contexts (Gross, 2015; Pekrun and Linnenbrink-Garcia, 2022). It can be conceptualized as an umbrella term involving cognitive, motivational, physiological, and expressive components, whereas emotion refers to more acute, situation-specific reactions that arise from appraisals of people, objects, or events (Ben-Eliyahu, 2019; Pham, 2007; Prinz, 2012; Scherer and Moors, 2019). Because learning involves more than the task itself, regulating the learning process, including learners’ emotional responses as they engage with instruction, is critical (Seufert et al., 2024).

Emotions vary along valence (pleasant vs. unpleasant) and arousal (activating vs. deactivating) and can be adaptive (helpful) or maladaptive (unhelpful) depending on context (Gross, 2015; Pekrun, 2006; Pekrun and Perry, 2014; Russell, 2003). For example, moderate and manageable levels of stress or state anxiety may support motivation for some learners under certain conditions, illustrating that neither positive nor negative emotions are inherently productive or unproductive (Marroquín, 2011; Plass and Kaplan, 2016). These contextual nuances underscore why the regulation of emotion, rather than its simple presence or absence, is central for learning.

Affect can bias cognition and behavior in ways that either support or hinder goal pursuit (Blanchette and Richards, 2010; Gendolla, 2000; Siemer, 2001). Emotion regulation, a form of self-regulation, refers to processes through which individuals recognize, evaluate, and modify emotional responses to function optimally and achieve goals (Aldao et al., 2010; Ben-Eliyahu, 2019; Gross, 2015; Gross and John, 2003; Harley et al., 2019; Koole, 2009, 2010; Marroquín, 2011). These processes range from effortful, deliberate strategies to more automatic adjustments and are continuously engaged throughout learning activities (Huang et al., 2024; Koole and Kuhl, 2007; Mauss et al., 2007; Ochsner and Gross, 2005, 2008).

According to skill acquisition theory, cognitive processes that are initially effortful and resource-intensive become progressively more automatic and less demanding of working memory through deliberate, repeated practice (DeKeyser, 2020). Applied to emotion regulation, this suggests that regulatory strategies such as cognitive reappraisal, though initially requiring conscious cognitive effort and therefore imposing some load on working memory, may become increasingly automatic and efficient as learners engage with them consistently over time (Schwonke, 2015; Brockbank and Feldon, 2024). Within a CLT framework, this progression toward regulatory automaticity carries an important implication, in that, as regulatory strategies are assimilated into learners’ cognitive routines, the working memory resources required to deploy them diminish, potentially reducing the total load imposed during learning and freeing capacity for task-relevant processing (Sweller et al., 2019; Seufert et al., 2024).

Through such strategies, learners can exert some control over transient emotional experiences, taking an active role in shaping their learning environments and outcomes (Ben-Eliyahu, 2019; Koole, 2010). Emotion regulation is therefore implicated in many goal-directed cognitions and behaviors during learning, aligning closely with processes that CLT identifies as central to effective task engagement (Ben-Eliyahu and Linnenbrink-Garcia, 2013, 2015). When emotions become unhelpful, such as when anxiety or frustration consume working memory resources, adaptive regulation can reduce interference with task-relevant processing and thereby lower perceived cognitive load (Gross, 2015; Sweller et al., 2019).

Among regulatory strategies, cognitive reappraisal appears especially promising for mitigating load-related difficulties in CLT-relevant contexts (Brockbank and Feldon, 2024; Plass and Kalyuga, 2019; Seufert et al., 2024; Willner et al., 2022). Because emotional responses follow from appraisals, reappraisal targets the interpretation of learning situations so that emotions become more adaptive or more closely aligned with learners’ goals (Gross, 2015; Koole, 2010; McRae, 2016). By reframing a demanding task as a challenge rather than a threat, for instance, learners may experience less debilitating anxiety and free more working memory capacity for germane processing.

Empirically, cognitive reappraisal is associated with more favorable psychological states and mental health, and it can modulate cognitive responses to support desired emotional experiences during learning (Huang et al., 2024; McRae, 2016). Within a CLT framework, this suggests that reappraisal may help reduce perceived extraneous load by altering how learners construe instructional demands, even when task complexity itself is unchanged (Plass and Kalyuga, 2019; Seufert et al., 2024). Thus, reappraisal offers a theoretically grounded mechanism through which learners can actively manage the affective component of load-intensive tasks.

Despite these links, CLT research has seldom examined how affect and emotion regulation interact with cognitive load during learning (Brockbank and Feldon, 2024; Hawthorne et al., 2019, 2025; Plass and Kalyuga, 2019). This gap persists even though emotions are acknowledged as potentially constraining working memory by competing with task-relevant processes, thereby increasing load and diminishing transfer (Sweller et al., 2019). Emerging work at the intersection of CLT and self-regulation frameworks highlights the need to integrate emotion regulation into models of cognitive load, positioning the current study to investigate how affect and regulatory strategies like cognitive reappraisal shape learners’ load perceptions and learning outcomes (de Bruin et al., 2020; de Bruin and van Merriënboer, 2017; Seufert, 2018; Seufert et al., 2024). Contributing to this literature, this study explores the relationships between cognitive load, affect, and regulatory strategies. We anticipate these results will further support the integration of affective and regulatory elements within CLT. More specifically, beyond cognitive reappraisal and expressive suppression, a broader representation of regulatory strategies is anticipated from qualitative findings.

## 4 Materials and methods

### 4.1 Research design

Using a convergent parallel mixed methods design (Creswell, 2013), both quantitative and qualitative associations between affective constructs, including use of emotion regulation strategies, and differences in cognitive load and learning were explored within a descriptive longitudinal study. Participants were self-selected and participation was voluntary.

Quantitatively, a multivariate latent state approach combined with random intercept cross-lagged panel models (RI-CLPM) approach (Hamaker, 2023) was used to examine how each variable changes over time and the extent to which each variable predicts others across time, thus enabling differentiation between bidirectional effects (affect → load vs. load → affect) and between-person versus within-person variance (see also Feldon et al., 2024).

Qualitatively, the study employed grounded theory, a qualitative research methodology that involves a continuous, iterative interplay between data collection and analysis to produce a conceptual framework that is firmly grounded in empirical reality (Corbin and Strauss, 1990). Hence, this study used both *a priori* and open-ended coding to distill categories of participants’ experiences related to mental effort, cognitive load, motivation, and emotion regulation. Qualitative findings provided insight into learner experiences accompanying the dynamic relationships between quantitative measures.

### 4.2 Context and sample

Data collection took place at a public R1 university in the western United States during an introductory undergraduate statistics course over 8 weeks. Mathematical material has historically been associated with elevated cognitive load and a range of learner emotions (Boekaerts, 1997; Faust, 1996; Sweller, 1988), providing an optimal context for studying the proposed constructs.

The sample consisted of *N* = 144 undergraduate students (80% female, 20% male; 95% White, 1% Asian, 4% other; 6% Hispanic/Latino). Forty-six percent of participants were freshman, 32% sophomore, 20% junior, and 2% senior or beyond.

### 4.3 Data collection measures

#### 4.3.1 Quantitative

Math content knowledge was assessed utilizing both (1) participants’ ALEKS placement scores (Assessment and Learning in Knowledge Spaces; McGraw Hill, 2025), and (2) participants’ first and second exam scores. The 40-item Attitude toward Mathematics Inventory-short (ATMI-short; Lim and Chapman, 2013) was administered pre- and post-test to assess participants’ confidence (i.e., self-efficacy), motivation, value, and enjoyment toward mathematics.

Cognitive load was assessed weekly using an adapted 9-point Likert scale survey (Krieglstein et al., 2023) measuring participants’ perception of mental effort investment, material difficulty, and comprehension ease during weekly modules and assignments.

Additionally, the cognitive reappraisal and expressive suppression items from the Emotion Regulation Questionnaire (ERQ; Gross and John, 2003) were administered weekly to assess participants’ use of cognitive reappraisal (i.e., reassessing or reconsidering an emotional response for a more favorable outcome) and expressive suppression (i.e., inhibiting or blocking an emotional response expressively or behaviorally; see Gross, 2015; Huang et al., 2024).

Grounded in expectancy-value-cost theory (Barron and Hulleman, 2015), items adapted from Flake et al. (2015) and Kosovich et al. (2015) measured self-efficacy (i.e., ability beliefs) and motivational costs, including task-related effort cost, task-unrelated effort cost, loss of valued alternatives, and emotional/psychological cost (including overwhelm).

#### 4.3.2 Qualitative measures

Semi-structured interviews were conducted with randomly selected participants (*N* = 21; 10 male, 11 female) at two time points across 8 weeks (*N* = 42 interviews total, approximately 4 weeks apart). Interviews explored how affective constructs influence learning, emotion regulation strategies used, motivational factors, and conditions contributing to both unproductive and manageable cognitive load. Interviews were video recorded, transcribed, and de-identified.

### 4.4 Procedures

Students were self-selected from introductory Statistics courses and participation was voluntary. Course instructors were not informed of participating versus non-participating students. The study proceeded in three phases:

Phase 1 (Pre-test): Baseline measures included math content knowledge (ALEKS scores, first exam) and ATMI-short. Based on these data, 21 students were selected for interviews.

Phase 2 (Data Collection): Participants completed weekly surveys (cognitive load, ERQ, expectancy/motivational costs) following module completion (weeks 2–7). Semi-structured interviews with selected students were also conducted during this phase.

Phase 3 (Post-test): Second exam scores, post-test ATMI-short administration, interview coding and analysis, and final quantitative analyses were completed.

### 4.5 Data analysis

#### 4.5.1 Quantitative analysis

**FIGURE 1:**
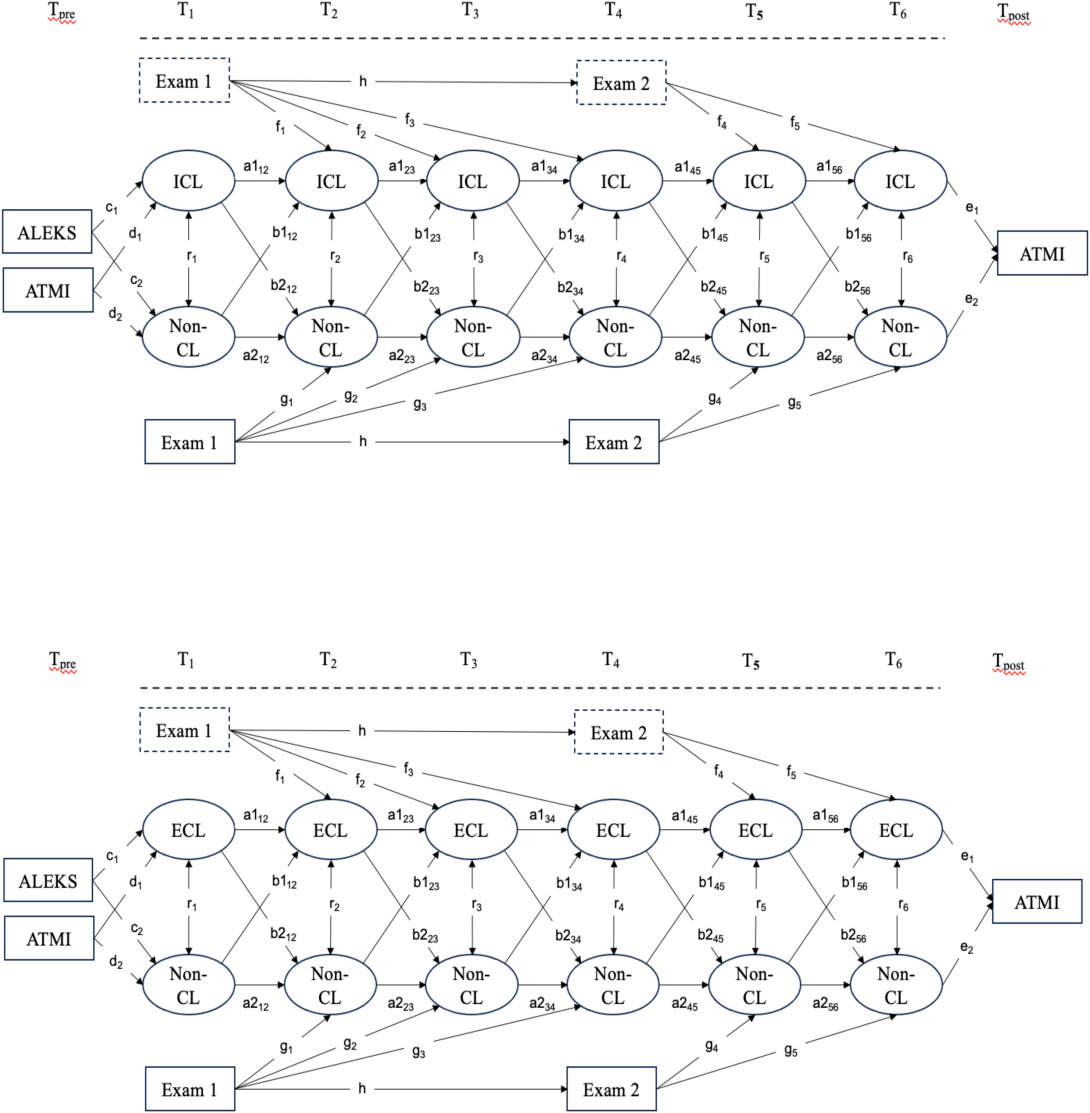
Latent state random-intercept cross-lagged panel models (LS-RI-CLPM) for ICL and ECL. Multivariate analyses with latent state RI-CLPM models were conducted to examine the relationship among cognitive load and the other variables. Each of the non-cognitive load variables (i.e., cognitive reappraisal, expressive suppression, motivational cost, emotional cost, and expectancy) can be put in place of Non-CL to include ICL or ECL (respectively) with the variable of choice (e.g., ICL with cognitive reappraisal, ICL with expressive suppression, etc.) For within and across time relationships, a = autoregression; b = cross-lagged regression; r = within time correlation; c, d, e = the relationships between the given variable (i.e., cognitive reappraisal, expressive suppression, etc.) and ALEKS pre-test, ATMI pre-test, and ATMI post-test, respectively. Further, f = regression paths between exam scores and non-cognitive load variables (i.e., cognitive reappraisal, expressive suppression, etc.), g = regression paths between exam scores and ICL/ECL, and h = regression path between Exam 2 and Exam 1.

To begin to understand the relationships among cognitive load and other variables, multivariate latent state RI-CLPM analyses (see Figure 1) using Mplus 8.10 with maximum likelihood estimation and robust standard errors (i.e., use of a sandwich estimator to account for non-normality, missingness, and within-section clustering) were conducted. These analyses typically are sometimes used to evaluate causal dynamics, yet such interpretation in the present study should be cautioned due to limitations in sample size and model complexity. Strong measurement invariance was imposed; state factor loadings and intercepts were constrained equal over time. This allowed us to examine mean-level change while constraining measurement properties to be statistically equivalent over time and was provisional given that formal invariance testing was not feasible due to quantitative model complexity. For this reason, the quantitative findings should be cautiously interpreted as preliminary to inform future work.

Given the sample size, two constructs were included per model: extraneous cognitive load (ECL) or intrinsic cognitive load (ICL) paired with one other variable (cognitive reappraisal, expressive suppression, motivational cost, emotional cost, or expectancy), yielding 10 analytic groupings. Structural relationships were estimated using within-time correlations, autoregressions across constructs, and first-order cross-lagged regressions between consecutive time points. Pre-test and post-test measures (ALEKS, ATMI, exam scores) were incorporated as regression paths to examine their associations with the time-varying constructs. All estimates are reported as standardized coefficients and are summarized in Tables 1, 2. Data was obtained and curated by the first author and statistical analyses were conducted by the third author. Minimum data coverage across variables was 53%. Due to relatively low sample size in combination with the large number of parameters, many constraints were imposed to estimate models (e.g., strong invariance across time, see online supplemental materials and appendices^1^). Model results are reported as standardized effects, where relevant.

**TABLE 1:** Standardized parameter estimates across all ECL models.

Parameter label	β (SE)				
	ECL, Cost	ECL, CR	ECL, EMC	ECL, EXP	ECL, EXS
*a1*_12_	0.57 (0.1)*	0.84 (0.16)*	0.55 (0.12)*	0.76 (0.19)*	0.84 (0.12)*
*a1*_23_	0.61 (0.4)	0.92 (0.11)*	0.6 (0.18)*	0.58 (0.16)*	0.88 (0.12)*
*a1*_34_	0.7 (0.08)*	0.49 (0.11)*	0.77 (0.03)*	0.69 (0.06)*	0.53 (0.13)*
*a1*_45_	0.82 (0.17)*	0.98 (0.15)*	0.74 (0.06)*	0.89 (0.06)*	1.03 (0.11)*
*a1*_56_	0.64 (0.18)*	0.74 (0.05)*	0.71 (0.09)*	0.89 (0.09)*	0.74 (0.07)*
*a2*_12_	0.95 (0.06)*	0.59 (0.16)*	0.72 (0.11)*	0.9 (0.17)*	0.85 (0.07)*
*a2*_23_	1.28 (0.15)*	0.8 (0.39)*	0.89 (0.03)*	0.87 (0.2)*	0.82 (0.08)*
*a2*_34_	0.99 (0.07)*	0.93 (0.3)*	0.95 (0.04)*	0.6 (0.13)*	1.15 (0.09)*
*a2*_45_	0.87 (0.13)*	0.96 (0.12)*	0.88 (0.05)*	0.97 (0.09)*	0.94 (0.03)*
*a2*_56_	0.99 (0.07)*	0.89 (0.12)*	0.85 (0.04)*	0.81 (0.08)*	0.96 (0.02)*
*b1*_12_	0.36 (0.13)*	−0.01 (0.11)	0.19 (0.07)*	−0.13 (0.16)	−0.01 (0.06)
*b1*_23_	0.34 (0.34)	0.11 (0.15)	0.27 (0.16)	−0.46 (0.21)*	0 (0.09)
*b1*_34_	−0.14 (0.01)*	−0.02 (0.05)	−0.07 (0.06)	0.1 (0.03)*	−0.15 (0.05)*
*b1*_45_	0.19 (0.12)	0.06 (0.12)	0.1 (0.1)	−0.29 (0.13)*	0.13 (0.08)
*b1*_56_	0.22 (0.26)	−0.04 (0.12)	0.18 (0.13)	0.14 (0.18)	0 (0.05)
*b2*_12_	−0.01 (0.07)	−0.17 (0.19)	0.03 (0.1)	−0.04 (0.06)	−0.02 (0.25)
*b2*_23_	−0.35 (0.17)*	−0.03 (0.1)	−0.05 (0.07)	0.04 (0.1)	−0.01 (0.06)
*b2*_34_	−0.04 (0.04)	0.09 (0.14)	0.05 (0.06)	−0.27 (0.17)	0 (0.06)
*b2*_45_	0.02 (0.17)	−0.28 (0.24)	−0.01 (0.1)	−0.06 (0.27)	−0.12 (0.09)
*b2*_56_	−0.01 (0.02)	−0.05 (0.07)	0 (0.1)	−0.14 (0.11)	−0.05 (0.08)
*c*_1_	−0.21 (0.2)	−0.24 (0.18)	−0.07 (0.07)	−0.24 (0.18)	−0.23 (0.18)
*c*_2_	−0.35 (0.08)*	−0.22 (0.31)	−0.27 (0.1)*	0.46 (0.12)*	−0.11 (0.5)
*d*_1_	−0.79 (0.15)*	−0.78 (0.13)*	−0.24 (0.11)*	−0.78 (0.14)*	−0.78 (0.13)*
*d*_2_	−0.75 (0.04)*	0.81 (0.14)*	−0.04 (0.13)	0.72 (0.11)*	0.36 (0.4)
*e*_1_	0.17 (0.33)	−0.05 (0.25)	0.04 (0.19)	0.23 (0.26)	−0.04 (0.22)
*e*_2_	−0.47 (0.08)*	0.17 (0.23)	−0.23 (0.1)*	0.51 (0.09)*	0.06 (0.19)
*f*_1_	0.11 (0.12)	0.15 (0.11)	0.03 (0.03)	0.13 (0.1)	0.14 (0.19)
*f*_2_	−0.25 (0.13)	−0.45 (0.3)	−0.08 (0.04)*	−0.05 (0.19)	−0.39 (0.26)
*f*_3_	0.85 (0.1)*	0.87 (0.08)*	0.26 (0.04)*	0.73 (0.09)*	0.94 (0.12)*
*f*_4_	−0.02 (0.24)	−0.05 (0.29)	−0.05 (0.1)	0.03 (0.17)	−0.12 (0.21)
*f*_5_	0.18 (0.09)*	0.22 (0.1)*	0.09 (0.05)	0.15 (0.14)	0.26 (0.1)*
*g*_1_	−0.14 (0.24)	0.62 (0.24)*	−0.03 (0.13)	0.46 (0.39)	0.16 (0.51)
*g*_2_	0.18 (0.15)	−1.02 (0.37)*	0.06 (0.02)*	0.14 (0.47)	0.43 (0.41)
*g*_3_	0.13 (0.13)	0.1 (0.35)	−0.02 (0.05)	0.3 (0.19)	−0.68 (0.41)
*g*_4_	0.26 (0.22)	0.32 (0.2)	0.08 (0.05)	−0.13 (0.11)	0.04 (0.31)
*g*_5_	−0.02 (0.06)	−0.06 (0.3)	−0.06 (0.01)*	0.09 (0.15)	0.11 (0.14)
*h*	0.97 (0.02)*	0.97 (0.02)*	0.49 (0.13)*	0.97 (0.02)*	0.97 (0.02)*
*r*_1_	0.49 (0.1)*	−0.05 (0.15)	0.47 (0.11)*	−0.19 (0.19)	0.1 (0.12)
*r*_2_	0.67 (0.1)*	−0.01 (0.2)	0.33 (0.04)*	−0.37 (0.08)*	−0.08 (0.07)
*r*_3_	0.44 (0.19)*	0.12 (0.04)*	0.16 (0.24)	−0.19 (0.08)*	0.25 (0.24)
*r*_4_	0.65 (0.16)*	0.3 (0.19)	0.38 (0.24)	0.04 (0.19)	0.38 (0.12)*
*r*_5_	0.15 (0.28)	0.26 (0.12)*	0.18 (0.09)*	−0.36 (0.27)	0.14 (0.45)
*r*_6_	−0.13 (0.14)	0.14 (0.18)	−0.11 (0.23)	−0.45 (0.24)	0.06 (0.26)

*P < 0.05; Parameter labels in the table match parameter labels in Figure 1 and should be interpreted alongside Figure 1. ECL, extraneous cognitive load; ICL, intrinsic cognitive load; Cost, motivational cost; EMC, emotional cost; EXP, expectancy for success; CR, cognitive reappraisal; EXS, expressive suppression.

**TABLE 2:** Standardized parameter estimates across all ICL models.

Parameter label	β (SE)				
	ICL, Cost	ICL, CR	ICL, EMC	ICL, EXP	ICL, EXS
*a1*_12_	0.69 (0.19)*	0.76 (0.15)*	0.41 (0.07)*	0.42 (0.11)*	0.79 (0.09)*
*a1*_23_	0.39 (0.11)*	0.74 (0.2)*	0.32 (0.07)*	0.48 (0.1)*	0.81 (0.2)*
*a1*_34_	0.81 (0.28)*	0.64 (0.27)*	0.77 (0.22)*	0.8 (0.13)*	0.75 (0.29)*
*a1*_45_	0.82 (0.24)*	1.06 (0.21)*	0.67 (0.09)*	0.79 (0.12)*	0.99 (0.3)*
*a1*_56_	0.65 (0.15)*	0.63 (0.11)*	0.7 (0.09)*	0.88 (0.13)*	0.65 (0.13)*
*a2*_12_	0.79 (0.21)*	0.67 (0.21)*	0.64 (0.1)*	0.84 (0.13)*	0.88 (0.1)*
*a2*_23_	1.04 (0.02)*	0.76 (0.43)	0.91 (0.02)*	0.51 (0.07)*	0.81 (0.11)*
*a2*_34_	1.15 (0.15)*	0.89 (0.32)*	0.95 (0.04)*	0.61 (0.11)*	1.15 (0.21)*
*a2*_45_	0.86 (0.09)*	1.02 (0.05)*	0.91 (0.15)*	0.73 (0.31)*	0.96 (0.07)*
*a2*_56_	1.07 (0.03)*	0.9 (0.13)*	0.83 (0.05)*	0.72 (0.16)*	0.97 (0.03)*
*b1*_12_	0.13 (0.15)	0.06 (0.15)	0.17 (0.07)*	−0.08 (0.14)	−0.16 (0.09)
*b1*_23_	0.57 (0.03)*	0.02 (0.14)	0.58 (0.06)*	−0.4 (0.16)*	0.13 (0.15)
*b1*_34_	−0.05 (0.16)	0.24 (0.16)	−0.05 (0.13)	0.07 (0.12)	−0.06 (0.1)
*b1*_45_	0.2 (0.12)	−0.24 (0.1)*	0.23 (0.09)*	−0.06 (0.14)	0.11 (0.16)
*b1*_56_	0.04 (0.16)	0.03 (0.15)	0.06 (0.1)	0.29 (0.19)	−0.07 (0.16)
*b2*_12_	0.2 (0.21)	0.06 (0.13)	0.28 (0.06)*	0.07 (0.04)	0.17 (0.28)
*b2*_23_	−0.08 (0.05)	0.04 (0.1)	−0.06 (0.03)*	−0.09 (0.1)	0 (0.09)
*b2*_34_	−0.18 (0.09)*	−0.06 (0.13)	0.04 (0.06)	−0.35 (0.17)*	0.01 (0.02)
*b2*_45_	0.11 (0.1)	0.07 (0.08)	−0.05 (0.24)	−0.03 (0.35)	−0.12 (0.08)
*b2*_56_	−0.1 (0.08)	0.04 (0.11)	0.04 (0.08)	−0.11 (0.16)	0.04 (0.08)
*c*_1_	−0.86 (0.14)*	−0.83 (0.14)*	−0.28 (0.09)*	−0.26 (0.09)*	−0.86 (0.13)*
*c*_2_	−0.33 (0.09)*	−0.2 (0.31)	−0.27 (0.11)*	0.26 (0.04)*	−0.06 (0.59)
*d*_1_	−0.1 (0.19)	−0.16 (0.18)	−0.07 (0.06)	−0.03 (0.06)	−0.15 (0.2)
*d*_2_	−0.75 (0.04)*	0.81 (0.13)*	−0.03 (0.13)	0.36 (0.11)*	0.39 (0.44)
*e*_1_	0.13 (0.14)	−0.03 (0.17)	0.05 (0.15)	0.15 (0.13)	−0.03 (0.11)
*e*_2_	−0.47 (0.07)*	0.15 (0.19)	−0.24 (0.09)*	0.39 (0.09)*	0.05 (0.18)
*f*_1_	0.07 (0.55)	0.11 (0.5)	0.01 (0.12)	0.01 (0.12)	−0.11 (0.32)
*f*_2_	0.25 (0.44)	0.19 (0.62)	0.02 (0.08)	0.11 (0.09)	0.32 (0.64)
*f*_3_	0.35 (0.35)	0.57 (0.09)*	0.07 (0.08)	0.06 (0.07)	0.34 (0.43)
*f*_4_	−0.2 (0.37)	−0.4 (0.48)	−0.1 (0.13)	−0.08 (0.11)	−0.34 (0.5)
*f*_5_	0.44 (0.33)	0.42 (0.27)	0.16 (0.09)	0.14 (0.09)	0.48 (0.32)
*g*_1_	−0.18 (0.26)	0.61 (0.29)*	−0.05 (0.15)	0.18 (0.14)	0.11 (0.58)
*g*_2_	0.04 (0.18)	−1.06 (0.34)*	0.06 (0.02)*	0.06 (0.13)	0.47 (0.45)
*g*_3_	0.18 (0.15)	0.07 (0.34)	−0.01 (0.05)	0.13 (0.02)*	−0.81 (0.44)
*g*_4_	0.22 (0.15)	0.2 (0.12)	0.08 (0.05)	−0.06 (0.05)	0.08 (0.32)
*g*_5_	−0.03 (0.05)	−0.09 (0.32)	−0.07 (0.01)*	0.04 (0.08)	0.1 (0.21)
*h*	0.97 (0.02)*	0.97 (0.02)*	0.49 (0.12)*	0.5 (0.13)*	0.98 (0.02)*
*r*_1_	0.22 (0.08)*	−0.12 (0.15)	0.24 (0.06)*	−0.5 (0.11)*	−0.12 (0.12)
*r*_2_	0.52 (0.13)*	0.06 (0.14)	0.37 (0.17)*	−0.35 (0.13)*	−0.21 (0.14)
*r*_3_	0.54 (0.11)*	−0.08 (0.05)	0.43 (0.21)*	−0.31 (0.12)*	−0.01 (0.18)
*r*_4_	0.88 (0.21)*	0.17 (0.15)	0.59 (0.33)	−0.12 (0.17)	0.32 (0.05)*
*r*_5_	0.34 (0.18)	0.19 (0.23)	0.27 (0.07)*	−0.36 (0.19)	0.29 (0.51)
*r*_6_	−0.14 (0.11)	−0.08 (0.14)	−0.25 (0.08)*	−0.4 (0.16)*	0.13 (0.35)

*P < 0.05; Parameter labels in the table match parameter labels in Figure 1 and should be interpreted alongside Figure 1. ECL, extraneous cognitive load; ICL, intrinsic cognitive load; Cost, motivational cost; EMC, emotional cost; EXP, expectancy for success; CR, cognitive reappraisal; EXS, expressive suppression.

#### 4.5.2 Qualitative analysis

Using grounded theory with deductive and inductive coding strategies (Corbin and Strauss, 1990), interviews were coded *a priori* for cognitive load (ICL and ECL), affect and emotion regulation, motivation and motivational costs, expectancies for success, and subjective task values. Emergent themes were also identified (e.g., intraindividual elements, broader regulatory strategies). The coding process yielded 26 codes organized into five categories: (1) cognitive load constructs (e.g., ICL vs. ECL, organizational strategies, multiple attempts to engage with learning material), (2) affective constructs (e.g., emotion, emotion regulation strategies), (3) motivational constructs related to cost (e.g., emotional/psychological cost, task-related effort cost, task-unrelated effort cost, loss of valued alternatives), (4) motivational constructs related to expectancy (e.g., high vs. low ability beliefs) and value (e.g., attainment value, intrinsic value, utility value), and (5) intraindividual elements (e.g., self-talk, autonomy, gratitude, taking breaks, social supports, breaking tasks into sub-goals). Completion and coding of all interviews was conducted by the first author.

## 5 Results

### 5.1 Quantitative findings

Due to the exploratory nature of this study, the following findings are preliminary in nature and represent promising progress toward furthering our knowledge of the dynamic interplay between cognitive load, affect, and regulatory strategies.

#### 5.1.1 Cognitive reappraisal and ECL

Multivariate RI-CLPM models were fit to the data^2^, assuming strong measurement invariance over time and across items. Standardized relationships within cognitive reappraisal and ECL were highly stable over time. Cross-lagged regressions did not reveal significant predictive effects between cognitive reappraisal and ECL overall, although ATMI pre-test scores were negatively associated with ECL (β = −0.69, *p* < 0.001) and positively associated with cognitive reappraisal 1 (β = 0.91, *p* < 0.001) at time 1. Cognitive reappraisal and ECL were modestly positively correlated within time at time 3 (*r* = 0.18, *p* = 0.009) and time 5 (*r* = 0.22, *p* = 0.01).

#### 5.1.2 Cognitive reappraisal and ICL

Relationships within cognitive reappraisal and ICL were also strongly stable over time. Cognitive reappraisal significantly predicted ICL from time 3 to 4 (β = 0.24, *p* = 0.02) and from time 4 to 5 (β = −0.17, *p* = 0.03), whereas ICL did not predict later cognitive reappraisal. Higher ALEKS pre-test scores were associated with lower ICL at time 1 (β = −0.89, *p* < 0.001), and higher ATMI pre-test scores were associated with higher cognitive reappraisal at time 1 (β = 0.90, *p* < 0.001). Within-time correlations between cognitive reappraisal and ICL were not significant.

#### 5.1.3 Expressive suppression and ECL

Expressive suppression and ECL each showed high within-construct stability. Expressive suppression significantly and positively predicted ECL from time 4 to 5 (β = 0.19, *p* = 0.02), with no other meaningful predictive or correlational effects between these constructs.

#### 5.1.4 Expressive suppression and ICL

Within-construct stability for expressive suppression and ICL was again high. Expressive suppression significantly and negatively predicted ICL from time 1 to 2 (β = −0.19, *p* = 0.02), while ICL did not predict subsequent expressive suppression. Higher ALEKS pre-test scores were associated with lower ICL at time 1 (β = −0.29, *p* = 0.001), and expressive suppression and ICL were positively correlated at time 4 (*r* = 0.19, *p* < 0.001).

#### 5.1.5 Motivational cost and ECL

Motivational cost and ECL demonstrated strong stability over time. Motivational cost significantly and positively predicted ECL from time 1 to 2 (β = 0.43, *p* = 0.02) and from time 4 to 5 (β = 0.32, *p* = 0.04), while negatively predicting from time 3 to 4 (β = −0.38, *p* = 0.001). From the other direction, ECL significantly and negatively predicted motivational cost from time 2 to 3 (β = −0.31, *p* = 0.04) ALEKS pre-test scores were negatively associated with motivational cost at time 1 (β = −0.34, *p* < 0.001), and ATMI pre-test scores significantly negatively predicted both motivational cost (β = −0.79, *p* < 0.001) and ECL (β = −0.74, *p* < 0.001) at time 1. ATMI post-test scores were negatively related to motivational cost (β = −0.51, *p* < 0.001), indicating that students with higher math interest reported lower motivational costs at post-test. Motivational cost and ECL were significantly and positively correlated during the first four time points (0.44 ≤ *r* ≤ 0.65, *p* ≤ 0.02), but not at times 5 and 6 (*p* > 0.05).

#### 5.1.6 Motivational cost and ICL

Motivational cost significantly and positively predicted ICL from time 2 to 3 (β = 0.33, *p* = 0.005), whereas ICL significantly and negatively predicted motivational cost over the same interval (β = −0.12, *p* = 0.03). As ALEKS pre-test scores increased, both ICL (β = −0.26, *p* = 0.003) and motivational cost (β = −0.13, *p* = 0.02) decreased at time 1. Higher ATMI pre-test scores were also associated with lower motivational cost at time 1 (β = −0.30, *p* = 0.005). ATMI post-test scores were negatively related to motivational cost (β = −0.24, *p* < 0.001), mirroring the pre-test pattern. Motivational cost and ICL were significantly and positively correlated within the first four time points (0.24 ≤ r ≤ 0.49, *p* ≤ 0.01), but not at times 5 and 6 (*p* > 0.05).

#### 5.1.7 Emotional cost and ECL

Emotional cost and ECL showed high within-construct stability. Emotional cost significantly and positively predicted ECL from time 1 to 2 (β = 0.21, *p* = 0.001), whereas ECL did not significantly predict later emotional cost. ALEKS pre-test scores were negatively related to emotional cost (β = −0.28, *p* = 0.004). ATMI post-test scores were also negatively associated with emotional cost (β = −0.18, *p* = 0.005), such that higher math interest was linked with lower emotional cost. Emotional cost and ECL were significantly positively correlated within time at time 1 (*r* = 0.49, *p* < 0.001), time 2 (*r* = 0.35, *p* < 0.001), and time 5 (*r* = 0.23, *p* = 0.009).

#### 5.1.8 Emotional cost and ICL

Emotional cost significantly and positively predicted ICL from time 1 to 2 (β = 0.14, *p* = 0.02) and from time 2 to 3 (β = 0.53, *p* < 0.001). ICL significantly predicted emotional cost from time 1 to 2 (β = 0.29, *p* < 0.001; positive) and from time 2 to 3 (β = −0.11, *p* = 0.003; negative). ALEKS pre-test scores were negatively associated with both emotional cost (β = −0.28, *p* = 0.01) and ICL (β = −0.29, *p* = 0.001) at time 1. Higher ATMI post-test scores were related to lower emotional cost (β = −0.18, *p* < 0.001). Emotional cost and ICL were significantly positively correlated within time at time 1 (*r* = 0.21, *p* < 0.001) and time 5 (*r* = 0.25, *p* < 0.001), and significantly negatively correlated at time 6 (*r* = −0.19, *p* = 0.03).

#### 5.1.9 Expectancy for success and ECL

Expectancy negatively predicted ECL from time 2 to 3 (β = −0.32, *p* = 0.003) and positively predicted ECL from time 3 to 4 (β = 0.17, *p* = 0.001), whereas ECL did not significantly predict expectancy. ALEKS pre-test scores (β = 0.26, *p* < 0.001) and ATMI pre-test scores (β = 0.38, *p* < 0.001) were both positively associated with expectancy at time 1, and ATMI post-test scores were similarly associated with higher expectancy (β = 0.32, *p* = 0.005). Within-time analyses showed that expectancy was negatively correlated with ECL at time 2 (*r* = −0.32, *p* < 0.001) and time 3 (*r* = −0.21, *p* = 0.009).

#### 5.1.10 Expectancy for success and ICL

Expectancy significantly and negatively predicted ICL from time 2 to 3 (β = −0.36, *p* = 0.01). In the reverse direction, ICL significantly positively predicted expectancy from time 1 to 2 (β = 0.11, *p* < 0.001) and significantly negatively predicted expectancy from time 3 to 4 (β = −0.29, *p* = 0.03). ALEKS pre-test scores were positively associated with expectancy (β = 0.27, *p* < 0.001) and negatively associated with ICL (β = −0.27, *p* < 0.001) at time 1, indicating that higher prior math performance was linked with higher expectancy and lower ICL. ATMI pre-test scores were also positively related to expectancy at time 1 (β = 0.33, *p* < 0.001). Expectancy was significantly negatively correlated with ICL at all six time points except time 4 (−0.51 ≤ *r* ≤ −0.30, *p* ≤ 0.001).

#### 5.1.11 Exam scores

Regression analyses between exam scores and the other variables yielded several notable effects. Exam 1 scores showed a dynamic relationship with cognitive reappraisal, with Exam 1 scores positively predicted cognitive reappraisal at Week 2 (the exam week) when ICL was included in the model (β = 0.61, *p* = 0.036) and when ECL was included (β = 0.62, *p* = 0.008), indicating that better performance on Exam 1 was associated with higher concurrent cognitive reappraisal. In contrast, Exam 1 scores negatively predicted cognitive reappraisal at Week 3 (the week after Exam 1) when ICL was controlled (β = −1.06, *p* = 0.002) and when ECL was controlled (β = −1.02, *p* = 0.005), suggesting a drop in reappraisal among higher-performing students the following week. All predictive relationships between Exam 2 and Exam 1 were positive and significant across models (0.49 ≤ β ≤ 0.97, *p* ≤ 0.001), reflecting stable exam performance over time.

### 5.2 Qualitative findings

Within qualitative findings, *a priori* categories are presented (e.g., cognitive load, emotion regulation, and motivational constructs), followed by findings from emergent categories of regulatory strategies. All excerpts represent participants’ transcribed responses. Minimal stylistic editing includes omitting excessive filler words (e.g., um, uh, like, so) or utterance portions not directly illustrating the targeted element (i.e., indicated by “…”) for length and ease of readability.

#### 5.2.1 Qualitative findings: cognitive load constructs

##### 5.2.1.1 Intrinsic cognitive load (ICL)

Interviewed students were asked questions regarding cognitive load factors, including distinctions between inherent task difficulty (i.e., ICL) and instructional factors (i.e., ECL). Their Statistics curriculum required mastery of unfamiliar vocabulary, novel concepts, and complex procedures. Students struggling with ICL-related learning experiences expressed it this way:

Student 3: *Some things are so hard… Like remembering all the different symbols and words, and the way they use the words… because a lot of them are so similar. It gets so confusing*.

Student 5: *All the concepts we have learned so far are all new to me… it can take me a while to understand a concept, especially if it is math related… This is a very fast-paced class and I might find it difficult to understand what we are currently learning if I am still trying to understand last week’s lessons*.

Another student expressed struggling with the complexity of the learning material: “It’s challenging – the content is challenging, in and of itself.” Yet other students acknowledged that while they found the learning material difficult, the challenge was manageable and meaningful learning was attainable. For example:

Student 7: *So far, I feel like I’ve been able to grasp and build upon everything*…*.I feel like I’ve been able to retain that knowledge and keep building on top of it… Obviously, I have to put like thought and work into it to understand things. But once I understand it, I remember*.

##### 5.2.1.2 Extraneous cognitive load (ECL)

Students interviewed identified multiple ECL sources, including technology complexity, instructor teaching style, lecture pace, visual clutter on slides, workspace constraints, and insufficient time between topics. Students often used words like “frustrated,” “stressed,” “discouraged,” “disappointed,” and “overwhelmed” to describe the challenges imposed by elements contributing to ECL. Further, students described how unhelpful (i.e., maladaptive) emotions such as frustration, overwhelm, anxiousness imposed unnecessary load, making it difficult for the students to focus on the task at hand.

Students explicitly noted the distinction between mental load imposed by extraneous factors (i.e., ECL) versus load imposed by inherent difficulty (i.e., ICL). When asked to rate the difficulty they were currently experiencing in their Statistics course on a scale from 1 (very, very easy) to 9 (very, very difficult), the following was shared:

Student 16: *I think the material itself is probably like a six out of ten difficulty – like a little bit harder than average, five being like average. I think how the material is presented and the lack of elaboration causes it to be like a nine*.

Technology imposed particular load. While appreciated, platform switches, timed exams, and interface complexity created frustration and distraction for students. Rapid lecture pace and information overload were also commonly cited:

Student 10: *It’s a lot of content - a lot very quickly… it’s definitely an information overload. There’s kind of like shot-gunning the information*.

Student 6: *Sometimes there’s so many screens in that lecture hall. Sometimes it can be overwhelming like, my gosh! There’s so many things in my view right now!*

Student 18: *Sometimes in the lecture hall… there’s just so many words and lots of bolding and underlining… it makes it really easy to get confused… And for me, when I get confused it’s really hard to like get back on track with the lecture*.

##### 5.2.1.3 Load-management strategies & structures

Regarding the myriad sources of cognitive load experienced during learning, participating students often talked about developing and using strategies or organizational structures to decrease mental load or make it feel more manageable. These strategies included breaking tasks into subgoals, using spacing and retrieval practice, taking restorative breaks, and leveraging support resources. Critically, students valued multiple attempts (retakes) with feedback for exams and assignments, which reduced anxiety and promoted learning from mistakes:

Student 7: *I approach tests with less anxiety because I see it more as like a “learning from failure” opportunity rather than like “if you fail, this is it”… I can learn from my mistakes and do better the next time. So emotionally, I would say I feel more composed*.

Student 30: *It makes me feel relieved… after I walk out of the testing center… I can take a breath of relief. And I’m like, okay – you do have a chance to retake it… It takes off a lot of mental stress*.

All students interviewed reported preferring limited retakes over unlimited attempts, viewing structured limits as promoting accountability and motivation.

Furthermore, most students noted that taking brief breaks was beneficial for managing both mental load and emotional overwhelm. This finding aligns with prior findings indicating working memory is dynamic in nature in that, upon depletion by mental effort, it recovers after rest (Chen et al., 2018). Brief breaks (e.g., physical activity, food, music, breathing) allowed emotional reset and cognitive recovery, enabling re-engagement:

Student 12: *When I feel overwhelmed, I usually will just take a break… I’ll just take a break and not do anything school related. And then I’ll come back to it*.

Student 31: *A lot of times, it takes just like a step back, coming to rethink things, or take a deep breath or something – and allowing myself to look at things from a different angle, or just like take a break, take a breather for a second*.

Student 29: *I definitely like pause for sure. Or like walk around – take a breather. Listening to music is a big one. I just like try to clear my mind*.

Many students emphasized the need for consistent review, repetition, and practice – strategies known to enhance learning (Kang, 2016; Mandler, 2014). As one student noted, “The easiest way for me to learn is repetition.” With this, taking the time to ensure adequate understanding was also relevant. Expressed this way:

Student 15: *But once we put it in practice, I think that’s what helps me the most – is just practice, practice, practice. That’s how Math is. Pretty much anything in life – applying it and trying to figure out what makes sense*.

#### 5.2.2 Qualitative findings: emotion regulation

To assess emotion regulation during interviews, participating students were invited to share how they managed or worked through unhelpful (i.e., often considered “negative”) emotions, including strategies used, if any.

##### 5.2.2.1 Cognitive reappraisal

Cognitive reappraisal (i.e., reframing challenging situations to view them as manageable), considered the most adaptive emotion regulation strategy, was the strategy students mentioned most (nearly twice as often as others). Students attributed reappraisal to freeing cognitive resources consumed by unhelpful emotion:

Student 3: *I think being able to shift how I’m thinking about something impacts a lot. I use it for everything. Because we all have those moments where we’re kind of irrational, and we’re like overwhelmed, and our emotions kind of get away from us…. There’s a lot of things that can seem overwhelming, but when you take a step back and you kind of break it down, it’s really not a big deal.…It generally goes better if I do have that moment to like reflect on what’s actually happening, and not what my emotions are telling me is happening*.

Student 7: *I think it’s important to not let negative emotion overwhelm you – but it is important to acknowledge that they exist. I think if you were to push it away, it’s only going to build up. It’s acknowledging that, assessing the situation, and then taking action – because if you sit with it for too long, then it gets harder to get out of that negative emotion. It’s both taking action in shifting your mind and taking physical action*.

Students who engaged in reappraisal reported facilitating action and problem-solving, thus reducing both perceived load and emotional burden. Students noted the importance of acknowledging feelings of discouragement, overwhelm, and intimidation during difficult learning, allowing oneself to “kind of get over those feelings of discomfort” by taking the first step of assessing the situation and then taking action – even micro-action – in a more helpful direction. Further, another student expressed feeling like “it’s a mindset thing when it comes to how much effort you’re putting in,” stating perspective “plays a huge part in learning.”

##### 5.2.2.2 Expressive suppression

Expressive suppression involves ignoring, avoiding, or stuffing emotions and is considered the most maladaptive emotion regulation strategy. Fewer participating students reported using expressive suppression and those who did acknowledged its ineffectiveness. Bottling emotions created mental “standstill,” hindering action:

Student 18: *I bottle a lot of my emotions up. when you’re feeling overwhelmed and you’re bottling emotions up, it puts you in a really weird standstill… I was trying to battle what I was feeling and then what I should be doing… I just was stuck*.

Student 22: *Yeah, my coping mechanism is to shut down. I don’t want to deal with it. I’d rather not have the feelings of stress and just kind of push it out of my way. Which can be really hindering at times*.

This association between “bottling” or “stuffing” strong maladaptive emotion and feeling mentally and physically “stuck” is particularly powerful, especially considering this experience adversely impacted students’ ability to take action. Students described links between cognitive reappraisal facilitating action via helping them move through and manage emotion and expressive suppression hindering action through bottling. Further, students described maladaptive (i.e., unhelpful) emotions as “demotivating,” stating these emotions shut them down and made them “not want to learn.” These students reported “suppressing” these emotions, which “made them get bigger.”

#### 5.2.3 Qualitative findings: motivation constructs related to cost

Throughout interviews, students articulated being both intrinsically and extrinsically motivated, depending on the context or circumstance. For example, many students reported enjoyment with course material, while also wanting a good grade. Throughout the interviews it was apparent, however, that while nearly all students expressed high value and enjoyment with learning in general, their love of and desire to learn was often adversely impacted by motivational costs (Barron and Hulleman, 2015; Flake et al., 2015).

##### 5.2.3.1 Emotional/psychological cost & overwhelm

Emotional and psychological costs associated with learning activities, interests, and goals were often keenly felt and expressed by students and were predominantly associated with maladaptive (i.e., “negative”) emotions. Feelings of stress, anxiety, frustration, confusion, discouragement, and fear of failure were common and often demotivating:

Student 18: *The frustration or the anxiety… makes me not want to learn. It just like shuts me off*.

Student 5: *Sometimes I can get frustrated with myself because I am not getting the right answer. When this happens, I tend to feel feelings of doubt that can make me less confident in myself and my abilities*.

When emotional and psychological costs became intense and unmanageable, the resulting overwhelm was particularly demotivating. This overwhelm often adversely impacted students’ abilities to engage with learning material effectively:

Student 16: *I feel overwhelmed for class like every day… It’s definitely demotivating*.

Student 10: *It’s just kind of like, I give up, and I don’t even know how to – it’s just overwhelming. I don’t know how to improve … and it’s really frustrating*.

Student 22: *I have had experiences before where, when it gets too much, I shut down in a sense*.

However, students who implemented cognitive reappraisal, organizational strategies, and took breaks effectively moderated these costs.

##### 5.2.3.2 Task-related effort cost & task-unrelated effort cost

Task-related effort cost involves is the negative perception of the amount of time, effort, or work inherent within a given task (Barron and Hulleman, 2015). Students reported that high intrinsic difficulty often required sustained mental effort:

Student 18: *It all comes down to the effort I have to put into it. It’s hard for me to mentally connect those pathways. So then, I have to put more effort into finding a new way for it to make sense*.

While effort itself was manageable for some, negative emotions accompanying difficulty amplified perceived cost for many students. Students regularly described expending intensive effort into learning complex material and completing assignments and exams.

Students also experienced negatively perceived effort required to complete tasks other than the primary task at hand. Similar to task-related efforts, task-unrelated efforts were also impacted by unhelpful or maladaptive thoughts and emotions.

##### 5.2.3.3 Opportunity cost/loss of valued alternatives

Opportunity cost encompasses the value of alternative activities that an individual gives up by choosing to engage in a particular task (Barron and Hulleman, 2015). Because there are only so many hours in each day, students had to choose where, and with what and whom, they would spend their time. Students consistently reported insufficient time, balancing multiple courses, jobs, and family:

Student 15: *I think it all comes down to time… I feel like I just can’t get enough of time*.

Student 30: *The workload I have in other classes, or the workload in this class as well, has been a struggle…and making sure I’m not putting too much effort into one and then not attending to the other*.

Student 22: *I gotta just recognize when I should be doing something and when I shouldn’t. I’m still trying to figure that out*.

This time scarcity created psychological pressure, positioning Statistics coursework as consuming disproportionate resources.

#### 5.2.4 Qualitative findings: expectancy and value

Throughout interviews, students’ expectancies for success (i.e., self-efficacy), along with their associated subjective values, directly influenced their motivation to engage and persist in learning tasks (Barron and Hulleman, 2015). Further, consistent with prior findings, the intrinsic value students placed on learning tasks was associated with higher expectancy beliefs (i.e., self-efficacy) as well as enhanced ability to manage higher cognitive demands during learning tasks (see Seufert et al., 2024).

##### 5.2.4.1 High vs. low expectancy for success

Higher expectancy beliefs typically lead to higher motivation, persistence, and resilience and are associated with stronger autonomy beliefs (i.e., beliefs in one’s ability to control one’s behavior and outcomes as well as influence one’s environment) (Bandura, 1997; Barron and Hulleman, 2015). Students interviewed often expressed strong ability beliefs, more than expected considering the levels of frustration, stress, and overwhelm articulated. Despite challenges and setbacks, most students expressed confidence in their ability to succeed through effort and persistence:

Student 26: *I know as long as I put in consistent effort, I haven’t ever really had anything that I couldn’t do*.

Student 5: *I know that if I can take the time and put the effort into class that is required, I know I can be successful in it*.

High expectancy was supported by prior success experiences and the belief that struggle is part of learning:

Student 27: *What makes me believe that I’ll be successful is just my past experiences, you know*.

Student 12: *I can understand that it’s a challenge, but I can overcome it. Because if I really think about it, then I can get the right answer or figure [it] out*.

Conversely, a small number of students reported low expectations within formal education, despite actively pursuing learning outside class:

Student 8: *I feel a little dumb – not because like I’m actually dumb, but I feel, like, deficient*.

Student 10: *I’m not very good at learning and that’s just the way it is. Like I know I’m not a super book-smart person, so I know it’s harder for me sometimes. But you know, I just have to work harder*.

##### 5.2.4.2 Subjective task values

Students most frequently reported valuing learning for its own sake (i.e., intrinsic value), particularly enjoying challenge and discovery:

Student 28: *I love learning and so I always love learning new things… it’s rewarding. I feel like I’m someone who can learn*.

Student 1: *Every time I learn something new, I just like feel proud. And like that always, like, makes me want to learn more*.

Student 1: *To me, learning is one of the most important things. I think every day I should be learning something new*…. *I should constantly be filling my mind*.

Students also reported prioritizing practical applicability, usefulness, or relevance of tasks (i.e., utility value):

Student 30: *What makes learning engaging for me? When it feels like I can apply it to real world situations*.

Student 12: *Like just me thinking about how I’ll be able to use this class for my degree… I’ll be using that hopefully in the future*.

For other students, performing well on a task or obtaining a desired outcome (i.e., attainment value) was prioritized. Attaining a “good grade” appeared to be valued by most students. While grades mattered, most students valued learning over grades alone. Some sought to prove capability to themselves:

Student 6: *To do well in this class… would kind of like prove something to me - that like I can get through a college Math class… And so, I think it has a lot of like internal worth to prove something to myself*.

#### 5.2.5 Qualitative findings: emergent regulatory strategies

During student interviews, additional themes of relevance emerged, often reflecting a strong sense of personal autonomy in learning, including a desire to do one’s best. Students expressed gratitude for opportunities to learn and for instrumental relationships in their lives that provided or scaffolded these learning opportunities. Students reported additional key strategies used to regulate emotions in a helpful manner. These include turning to social supports, indicating talking with family, friends, or peers provided perspective shifts and encouragement:

Student 7: *When I know that there’s people I can reach out to and like others I can work with, and different like perspectives or ideas, then it kind of creates this community of like, okay, I’ve got it*.

Student 15: *Just like talking about it with whoever – friends, family – just, I’m feeling this way. And then they’re like, yeah, you’ve got it, you’re fine*.

Recognizing others struggle with difficult emotions and learning how others manage or work through these emotions reportedly helped students cope better and learn more effectively. Further, students emphasized the importance of supportive social connections to both persistence and perseverance in learning.

Other adaptive regulatory strategies reported by students include use of organizational tools (e.g., planners, calendars), time management strategies (e.g., planning ahead, scheduling consistent study time), and taking small steps (e.g., breaking larger more complex tasks into smaller steps). Participating students emphasized breaking overwhelming tasks into “bite-sized pieces,” which reduced emotional overload and provided momentum:

Student 27: *I have to take it bit by bit. I break it down. Because I think about everything and then I almost shut down. But then, I can handle it because I just do a little bit at a time. That’s how I can get through it*.

Student 15: *I think breaking it into smaller chunks would generally make me happier*… *it helps to make me have like a more positive outlook on learning… your emotion toward learning will obviously be more positive if it’s something that’s more achievable*.

Positive inner dialogue (e.g., self-talk) also positively influenced emotion and motivation. Students linked positive self-talk to reappraisal:

Student 8: *My self-talk… plays a huge role because my self-talk are my thoughts and they are my emotions… it drives the way I behave*.

Student 26: *If I can keep it more positive, I’m definitely able to get through things faster, grasp topics better*.

Student 23: *I feel like [positive] self-talk can be a motivating factor…if it’s something I really got to crack down on…. A lot of times that self-talk is the thing that gets me moving*.

Additionally, students reiterated the restorative nature of brief breaks to replenish cognitive, emotional, and physical resources, enabling them to re-engage more effectively with learning tasks.

#### 5.2.6 Qualitative findings: summary

This study used both deductive and inductive coding strategies within a grounded theory framework (Corbin and Strauss, 1990). *A priori* codes included key concepts associated with cognitive load (ICL and ECL), affect and emotion regulation, motivation and motivational costs, expectancies for success, and subjective task values. Emergent codes pertaining to individuals’ self-regulatory efforts were also identified during qualitative analysis. During interviews, students’ experiences broadly illustrated multi-faceted examples of cognitive, affective, motivational, behavioral, and somatic regulatory strategies. Importantly, students did not employ regulatory categories in isolation. For example, reappraisal often co-occurred with positive self-talk and organizational planning. Taking a break often combined benefits of somatic recovery, affective diffusion, and motivational reset. This integrated approach appeared beneficial for reducing cognitive load from multiple, mutually reinforcing directions while students navigated complex, high-load learning contexts.

## 6 Discussion

This study examines the relationships between the cognitive load experienced in learning contexts and emotional states and motivational constructs, exploring interactions between load and psychosocial factors impacting well-being, including affect, motivational constructs, and regulatory strategies. Results indicate that emotion regulation may not be an “add-on” to cognitive load management, but a fundamental mechanism through which participants navigated demanding learning tasks.

### 6.1 Broadening cognitive load theory: the role of affect and regulation

Cognitive load theory has historically prioritized instructional and content factors (i.e., intrinsic and extraneous load) as the primary levers for optimizing learning (Sweller et al., 1998, 2019). This study provides novel exploratory quantitative and qualitative evidence suggesting cognitive load may be fundamentally shaped not only by task complexity and instructional design, but also by learners’ affective states, emotional costs, and deployed regulatory strategies. Findings of the current study may extend CLT by indicating affect and emotion regulation may not be peripheral to load dynamics, but potential mechanisms through which learners experience, interpret, and ultimately manage cognitive demands (see also Brockbank and Feldon, 2024; Hawthorne et al., 2025; Plass and Kalyuga, 2019).

Findings suggest cognitive load and emotion reciprocally influenced one another in bidirectional relationships, yet did so inconsistently over the course of learning. This suggests these relationships may be more or less important at different stages of the learning process. Higher emotional costs early in the semester were associated with increased load, while successful regulation (e.g., cognitive reappraisal, breaks, social support, and organizational strategies) moderated emotional cost and supported learning gains, as suggested by improved post-test ATMI scores. Students with more positive baseline math attitudes reported experiencing lower early load and cost perceptions.

Exam performance patterns further illustrate these dynamics. Higher-performing students on Exam 1 engaged in cognitive reappraisal during the exam week but reduced its use afterward, while lower performers increased reappraisal post-exam, possibly to re-motivate effort. By Exam 2, successful regulation was evident across all time-varying constructs, with Exam 2 scores positively predicting all constructs across models, indicating that emotion regulation effectiveness potentially supported not only immediate performance but sustained subsequent engagement, positioning regulated affect as a possible lever for managing perceived load.

Taken together, these patterns suggest that affective dispositions and emotional burdens may shape how learners perceive and experience cognitive demands, even before task engagement. This is consistent with theoretical models positing affect and cognition as deeply intertwined processes (see also Ben-Eliyahu, 2019; Blanchette and Richards, 2010; Brockbank and Feldon, 2024; Hawthorne et al., 2025).

### 6.2 Emotion regulation as a load-management mechanism

Within this study, students who actively regulated emotion – through cognitive reappraisal, organization strategies, brief breaks, or social connection – reported not only lower perceived load but also higher motivation, resilience, and learning outcomes. Findings indicate emotion regulation and cognitive load management may be interconnected processes (Seufert, 2018; Seufert et al., 2024).

Among emotion regulation strategies, cognitive reappraisal emerged as the most adaptive, appearing nearly twice as frequently in student interviews as other approaches and consistently associated with productive action, reduced load perception, enhanced motivation, and problem-solving. Students’ own accounts reveal the mechanism of cognitive reappraisal allowing learners to reframe emotionally charged situations, viewing overwhelm as a temporary state and difficulty as challenge rather than threat, thereby freeing cognitive resources consumed by maladaptive affective states, and enabling action toward learning goals (see Gross, 2015).

Within-time correlations showed that students reporting higher perceived ECL also more frequently engaged in cognitive reappraisal, suggesting adaptive regulation in response to heightened load. Organizational strategies (e.g., planning, breaking tasks into sub-steps), brief breaks, and social support were also highly valued by students as regulatory measures, and multiple attempts with feedback were reported to reduce anxiety and promote learning by reframing performance setbacks as learning opportunities rather than failures, thereby reducing perceived state anxiety and promoting persistence. These dynamic findings could be useful for identifying and providing adaptive scaffolding in the learning process appropriate for different students.

In contrast, expressive suppression reportedly created perceived mental paralysis and intensified load-related difficulties over time. Suppression negatively predicted ICL early but positively predicted ECL later, reflecting the ineffectiveness of expressive suppression in reducing emotional burden and its tendency to consume additional cognitive resources through thought/emotion suppression and rumination. Within CLT, this is consistent with maladaptive affect imposing extraneous load by competing for limited working memory resources, a mechanism consistent with Plass and Kalyuga’s (2019) conceptualization of emotion as ECL.

### 6.3 Regulatory strategy use and working memory: efficiency and automaticity

Findings from this study indicate self-regulatory strategies – particularly emotion regulation strategies – may hold promising potential in moderating the relationships between cognitive load and affective variables, including motivational constructs. Because self-regulation is an “effortful process,” it “presupposes motivation” and can influence affect (Efklides, 2011, p. 6; see also Efklides and Schwartz, 2024). Thus, emotion regulation is foundational within all other self-regulatory strategies (e.g., behavioral, motivational, cognitive). Self-regulation in learning is typically influenced by (1) level of task difficulty, (2) learners’ cognitive resources, and (3) level of cognitive load (Seufert et al., 2024). Through this lens, perceived cognitive load increases with task difficulty due to higher demand on cognitive resources needed for the learning task. Seufert and colleagues describe this process in terms of an inverted U-shaped relationship wherein learners typically use fewer regulatory strategies when task difficulty is perceived as too easy or too difficult (see also Middleton and Midgley, 2002; Turner and Meyer, 2004). Because cognitive load is directly linked to both task difficulty and self-regulatory demands, it serves as an “important predictor for self-regulatory activities” (Seufert et al., 2024, p.19).

Prior studies indicate learners may respond to increasing task difficulty with increased regulatory strategy use. Indeed, de Bruin and colleagues (de Bruin et al., 2020; de Bruin and van Merriënboer, 2017) position the need to integrate self-regulatory processes and strategies within CLT, noting the complex interplay between increasing availability of information (due to technological advances) and the associated cognitive demands inherent within such advancing availability. As learners devote mental effort to the learning task at hand, they may need to invest additional effort in self-regulating learning processes, compounding cognitive load (de Bruin et al., 2020).

An important consideration, however, is that because of working memory limitations, demand imposed by both the learning task and accompanying self-regulatory processes may ultimately exceed working memory capacity, resulting in cognitive overload (Moos and Azevedo, 2008; Seufert et al., 2024; Van Gog et al., 2011). Thus, while regulatory strategies are intended to reduce cognitive load, the use of such strategies, particularly with high-difficulty tasks, may also utilize limited working memory resources. This applies especially when regulatory strategies are novel (i.e., new or not consistently used by the learner). However, as discussed above, skill acquisition research (DeKeyser, 2020) suggests the possibility that regulatory strategies consistently engaged in the learning process may become increasingly automatic, reducing strain on cognitive resources (Brockbank and Feldon, 2024; Schwonke, 2015). During this assimilation process, fewer and fewer cognitive resources may be needed for regulatory strategies, potentially moving learners toward more regulated (i.e., moderate) mental load. Thus, optimizing cognitive load during learning via assimilated strategy use may be highly beneficial within self-regulated learning (de Bruin et al., 2020; de Bruin and van Merriënboer, 2017; Efklides, 2011).

In this study, quantitative data revealed that higher cognitive reappraisal use was associated with higher perceived load within time (concurrent with load) yet lower load across time. This pattern aligns with schema acquisition theory in that initial strategy deployment is effortful and resource-intensive, but through repeated use and integration, strategies can become increasingly automatic and efficient (Brockbank and Feldon, 2024; Meylani, 2024; Schwonke, 2015). Implications for CLT may point to adaptive regulatory strategy automaticity potentially reducing overall load by managing affective interference without imposing substantial cognitive cost. Although our data cannot directly test regulatory automaticity, the observed pattern, in which concurrent reappraisal use was associated with higher within-time load but lower across-time load, appears consistent with this interpretation and warrants future investigation.

### 6.4 Implications for CLT and practice

#### 6.4.1 Theoretical integration

Study findings suggest the need for CLT’s theoretical boundaries to be enlarged to formally integrate affective, motivational, and regulatory processes. Historically, CLT has regarded affect as either external to load dynamics or as a secondary outcome of load (Sweller et al., 2019; Brockbank and Feldon, 2024). This study indicates that affect and load may reciprocally influence one another and that regulation may serve as a critical linking mechanism. An expanded CLT could model how emotional costs contribute to perceived load, how load may potentially trigger maladaptive emotion requiring regulation, and how regulatory skill development over time may reduce load efficiency losses. As Pekrun (2006) noted, “lack of theoretical integration. has to be overcome if cumulative theoretical and empirical progress is to be made” (p. 330). This work contributes empirically and theoretically to that integration.

To further integrate relevant affective and regulatory elements within CLT, the authors propose the conceptualization of affective cognitive load (ACL) as a subtype of ECL within the existing CLT framework. Because ECL can originate from a myriad of sources, delineating affect-based ECL may be of benefit in identifying potential mitigators, including regulatory strategies. Accordingly, we propose that ACL refer to the portion of extraneous load imposed on working memory that arises from affective states (including emotion), affective appraisals, and affect-related regulatory processes that are engaged during learning and instruction. Consistent with CLT’s core assumption that working memory capacity is limited (Sweller et al., 1998, 2019; Paas et al., 2003; van Merriënboer and Sweller, 2005), ACL captures the extent to which emotions (e.g., anxiety, frustration, overwhelm), motivational appraisals (e.g., perceived cost, self-efficacy), and affect-driven attentional processes consume, redirect, or modulate working memory resources while learners engage with instructional material. Thus, ACL would conceptually originate from learners’ maladaptive affective responses to learning conditions and their ongoing appraisal of effort, control, and value. Maladaptive affective states and poorly regulated emotional responses may increase total load and exacerbate ECL by diverting resources away from task-relevant processing. In this way, ACL may be conceptualized as a dynamically interacting component of ECL that reflects the inseparability of cognition, affect, and motivation within human cognitive architecture (Plass and Kalyuga, 2019; Kalyuga, 2011; Sweller et al., 2019).

#### 6.4.2 Pedagogical implications

If emotion regulation and cognitive load are potentially linked, then instruction should explicitly address both. This involves (1) designing learning environments and instructional sequences to minimize extraneous load, as CLT prescribes, while also (2) equipping learners with explicit guidance in emotion regulation strategies, including explicit instruction, modeling, cuing, and specific feedback, so learners can manage affective interference during cognitively demanding tasks. Structural supports such as multiple attempts with feedback, task decomposition, organizational tools, and social interaction opportunities may enable regulatory skill development. Metacognitive training that teaches learners to monitor their emotional and cognitive states alongside strategy use may enhance transferability and autonomy (Efklides and Schwartz, 2024).

#### 6.4.3 Supporting learner autonomy

A broader implication concerns agency, in that, because regulatory strategies are learner-controlled rather than instruction-dependent, they empower students to take active responsibility for managing their learning experience. Findings from this study indicate that students who engaged adaptive strategies reported not only lower perceived load but also higher expectancy for success, greater engagement, and improved resilience in the face of difficulty. Fostering this agentic, self-regulated approach to learning, where learners are encouraged to notice, name, and skillfully manage their emotional and cognitive experience, aligns with humanistic learning principles and may be particularly valuable in challenging, high-stakes domains such as STEM-related learning (Ben-Eliyahu, 2019; Ben-Eliyahu and Linnenbrink-Garcia, 2015; Seufert, 2018).

Because learning is ultimately an active state involving motivation and engagement, factors that negatively impact action tendencies would have deleterious effects on learning. Explicit instruction in emotion regulation strategies, combined with thoughtful instructional design that minimizes extraneous load, may offer a promising pathway toward creating learning experiences that are not only cognitively efficient but also emotionally sustainable and personally empowering.

### 6.5 Discussion summary

This study endeavors to extend CLT by providing exploratory evidence that cognitive load may be shaped not only by instructional and task-related factors, but also by learners’ affective states, emotional costs, and regulatory strategies. Thus, findings position affect and regulation as potentially integral components of cognitive load dynamics rather than peripheral influences (Brockbank and Feldon, 2024; Plass and Kalyuga, 2019; Seufert et al., 2024). Quantitative and qualitative findings revealed bidirectional, time-varying relationships between affect and cognitive load, with higher emotional costs early in learning predicting increased perceived load, while adaptive affect and effective emotion regulation appeared to buffer load and support learning. Emotion regulation, particularly cognitive reappraisal, emerged as a promising load-management mechanism. Although emotion regulation may be initially effortful and associated with higher load, repeated use across time may be linked to reduced subsequent load, consistent with schema acquisition and increasing regulatory automaticity (Ben-Eliyahu, 2019; Gross, 2015; Schwonke, 2015; Seufert et al., 2024). In contrast, expressive suppression appeared to exacerbate extraneous load over time, underscoring its maladaptive nature. In this study, students flexibly combined cognitive, affective, motivational, behavioral, and somatic strategies to manage high-load contexts, suggesting that regulation operates as a coordinated, multi-system process. Collectively, these preliminary findings may support an expanded CLT framework that integrates affect, motivation, and regulation. These findings further highlight the instructional value of explicitly scaffolding emotion regulation and metacognitive monitoring to foster learner autonomy, resilience, and efficient load management (Efklides, 2011; Pekrun, 2006). Finally, to enable integration of affective and regulatory elements within CLT, the authors propose the conceptualization of affective cognitive load (ACL) as a subtype of ECL within the existing CLT framework.

## 7 Limitations and future directions

This study had several limitations. First, study enrollment fell short of optimal levels, resulting in insufficient statistical power to analyze all interactions in a single model. Accordingly, it is possible that important relationships between variables were not identified due to a lack of statistical significance. Second, although interviews provided rich insights into the experiences and strategies used by some participants, the number of interview participants represented less than 20% of the total study sample. Accordingly, the trends identified in the qualitative data may not be representative of participants from whom only quantitative data were collected. Third, participants represented a relatively narrow age group with limited demographic variability, which limits the generalizability of the findings. Further, because students self-selected, personal characteristics (i.e., motivational, behavioral) may have skewed findings relative to a sample more representative of the natural population. Fourth, the study’s descriptive (i.e., non-intervention) design prevents causal inferences from being drawn. While participants articulated their experiences of causality while articulating the perceived benefits of various self-regulation strategies, such evidence is only suggestive of possible impacts that require intervention studies to assess independently. Most sources of quantitative data similarly relied on self-reported perceptions collected in an anticipatory or *post hoc* manner, which may have produced different outcomes than other modes of data collection that are less susceptible to social desirability or halo effects. However, as the first study of its kind longitudinally integrating measures of cognition, motivation, and emotion regulation, it provides a promising foundation for future research.

Subsequent studies of the dynamic relationships between cognition, motivation, and emotion regulation would benefit from intervention-based designs to systematically manipulate levels and types of cognitive load. Similarly, interventions to enhance students’ ability to regulate emotions in learning environments would further clarify the prospective causal relationships among variables. Because both learners and instructors may similarly lack foundational regulatory understanding, future directions also include training instructors regarding regulatory strategies, including foundational knowledge of cognitive and affective processes, implementation of key strategies, and assessment of effectiveness, thus enhancing their ability to promote these skills among their students via intentional scaffolding during instruction.

## 8 Conclusion

While CLT has historically operated within predominantly cognitive boundaries, the findings of this study suggest that understanding the dynamics of cognitive load in learning requires attending to affective, motivational, and regulatory dimensions. Quantitative findings suggest reciprocal relationships between emotional costs and both ICL and ECL across 8 weeks. Qualitative findings illustrate how students intuitively deploy adaptive strategies (e.g., cognitive reappraisal, task decomposition, breaks, social support, positive self-talk) to adaptively manage the emotional load often accompanying cognitive demands.

Most importantly, this study explores emotion regulation as a potential fundamental mechanism through which learners navigate demanding learning tasks rather than an “add-on” to cognitive load management. Though these preliminary findings are exploratory and descriptive, further investigation into these relationships is merited, including interventions exploring the use of regulatory strategies to mitigate cognitive load and motivational costs. Current findings point toward a more integrated framework for understanding learning that encompasses cognition, affect, motivation, and self-regulation as mutually constitutive processes, including the proposal of ACL as a theoretically motivated subtype of ECL. Looking forward, empirical and theoretical work at the intersection of CLT and emotion regulation research may yield powerful insights into how learners develop resilience, autonomy, and effectiveness in the face of cognitive challenge.

## Data Availability

The raw data supporting the conclusions of this article will be made available by the authors, without undue reservation.

## Footnotes

1 Online supplemental materials and appendices are located at https://osf.io/3tkn4/overview?view_only=d0ed8dcf93694cb18ffcc58a272ea265

2 Given the small sample sizes, all except two models converged but were unable to estimate fit statistics due to the number of estimated parameters in the model, even after constraints were applied appropriately (see online supplemental materials for all quantitative model input and output). This is a limitation of the present modeling approach. In general, results demonstrate relationships that were also found in the separate qualitative analysis and should be replicated with larger sample sizes. No causal inference should be made regarding these findings.

## References

[B1] Aldao A. Nolen-Hoeksema S. Schweizer S. (2010). Emotion-regulation strategies across psychopathology: A meta-analytic review. *Clin. Psychol. Rev.* 30 217–237. 10.1016/j.cpr.2009.11.004 20015584

[B2] Baddeley A. (1992). Working memory: The interface between memory and cognition. *J. Cogn. Neurosci.* 4 281–288. 10.1162/jocn.1992.4.3.281 23964884

[B3] Bandura A. (1997). *Self-efficacy: The Exercise of Control*. New York, NY: Freeman.

[B4] Barron K. E. Hulleman C. S. (2015). Expectancy-value-cost model of motivation. *Psychology* 84 261–271. 10.1016/B978-0-08-097086-8.26099-6

[B5] Ben-Eliyahu A. (2019). Academic emotional learning: A critical component of self- regulated learning in the emotional learning cycle. *Educ. Psychol.* 54 84–105. 10.1080/00461520.2019.1582345

[B6] Ben-Eliyahu A. Linnenbrink-Garcia L. (2013). Extending self-regulated learning to include self-regulated emotion strategies. *Mot. Emot.* 37 558–573. 10.1007/s11031-012-9332-3

[B7] Ben-Eliyahu A. Linnenbrink-Garcia L. (2015). Integrating the regulation of affect, behavior, and cognition into self-regulated learning paradigms among secondary and post-secondary students. *Metacogn. Learn.* 10 15–42. 10.1007/s11409-014-9129-8

[B8] Blanchette I. Richards A. (2010). The influence of affect on higher level cognition: A review of research on interpretation, judgement, decision making and reasoning. *Cogn. Emot.* 24 561–595. 10.1080/02699930903132496

[B10] Boekaerts M. (1997). Capacity, inclination, and sensitivity for mathematics. *Anxiety Stress Coping* 10 5–33. 10.1080/10615809708249293

[B12] Brockbank R. B. Feldon D. F. (2024). Cognitive reappraisal: The bridge between cognitive load and emotion. *Educ. Sci.* 14:870. 10.3390/educsci14080870

[B13] Chen O. Castro-Alonso J. C. Paas F. Sweller J. (2018). Extending cognitive load theory to incorporate working memory resource depletion: Evidence from the spacing effect. *Educ. Psychol. Rev.* 30 483–501. 10.1007/s10648-017-9426-2

[B14] Corbin J. M. Strauss A. (1990). Grounded theory research: Procedures, canons, and evaluative criteria. *Qual. Sociol.* 13 3–21. 10.1007/BF00988593

[B15] Cowan N. (2001). Metatheory of storage capacity limits. *Behav. Brain Sci.* 24 154–176. 10.1017/S0140525X0161392X 11515286

[B16] Creswell J. W. (2013). *Steps in Conducting a Scholarly Mixed Methods Study. DBER Speaker Series 48.* Available online at: https://digitalcommons.unl.edu/dberspeakers/48

[B17] de Bruin A. B. van Merriënboer J. J. (2017). Bridging cognitive load and self-regulated learning research: A complementary approach to contemporary issues in educational research. *Learn. Instr.* 51 1–9. 10.1016/j.learninstruc.2017.06.001

[B18] de Bruin A. B. Roelle J. Carpenter S. K. Baars M. Efg-Mre. (2020). Synthesizing cognitive load and self-regulation theory: A theoretical framework and research agenda. *Educ. Psychol. Rev.* 32 903–915. 10.1007/s10648-020-09576-4

[B19] DeKeyser R. (2020). “Skill acquisition theory,” in *Theories in Second Language Acquisition*, eds VanPatten B. Williams J. (Mahwah, NJ: Lawrence Erlbaum), 83–104.

[B20] Eccles J. S. Wigfield A. (2023). Expectancy-value theory to situated expectancy-value theory: Reflections on the legacy of 40+ years of working together. *Motiv. Sci.* 9 1–12. 10.1037/mot0000275

[B21] Efklides A. (2011). Interactions of metacognition with motivation and affect in self-regulated learning: The MASRL model. *Educ. Psychol.* 46 6–25. 10.1080/00461520.2011.538645

[B22] Efklides A. Schwartz B. L. (2024). Revisiting the metacognitive and affective model of self- regulated learning: Origins, development, and future directions. *Educ. Psychol. Rev.* 36:61. 10.1007/s10648-024-09896-9

[B23] Faust M. W. (1996). Mathematics anxiety effects in simple and complex addition. *Math. Cogn.* 2 25–62. 10.1080/135467996387534

[B24] Feldon D. F. Brockbank R. Litson K. (2024). Direct effects of cognitive load on self- efficacy during instruction. *J. Educ. Psychol.* 116 1153–1171. 10.1037/edu0000826

[B25] Feldon D. F. Callan G. Juth S. Jeong S. (2019). Cognitive load as motivational cost. *Educ. Psychol. Rev.* 31 319–337. 10.1007/s10648-019-09464-6

[B26] Feldon D. F. Franco J. Chao J. Peugh J. Maahs-Fladung C. (2018). Self-efficacy change associated with a cognitive load-based intervention in an undergraduate biology course. *Learn. Instr.* 56 64–72. 10.1016/j.learninstruc.2018.04.007

[B28] Flake J. K. Barron K. E. Hulleman C. McCoach B. D. Welsh M. E. (2015). Measuring cost: The forgotten component of expectancy-value theory. *Contemp. Educ. Psychol.* 41 232–244. 10.1016/j.cedpsych.2015.03.002

[B30] Gendolla G. H. E. (2000). On the impact of mood on behavior: An integrative theory and a review. *Rev. Gen. Psychol.* 4 378–408. 10.1037/1089-2680.4.4.378

[B31] Gross J. J. (2015). Emotion regulation: Current status and future prospects. *Psychol. Inq.* 26 1–26. 10.1080/1047840X.2014.940781

[B32] Gross J. J. John O. P. (2003). Individual differences in two emotion regulation processes: Implications for affect, relationships, and well-being. *J. Pers. Soc. Psychol.* 85 348–362. 10.1037/0022-3514.85.2.348 12916575

[B33] Hamaker E. L. (2023). The within-between dispute in cross-lagged panel research and how to move forward. *Psychol. Methods*. 10.1037/met0000600 [Epub ahead of print]. 37902677

[B34] Harley J. M. Pekrun R. Taxer J. L. Gross J. J. (2019). Emotion regulation in achievement situations: An integrated model. *Educ. Psychol.* 54 106–126. 10.1080/00461520.2019.1587297

[B36] Hawthorne B. S. Slemp G. R. Vella-Brodrick D. A. Hattie J. (2025). The relationship between positive and painful emotions and cognitive load during an algebra learning task. *Learn. Individ. Differ.* 117:102597. 10.1016/j.lindif.2024.102597

[B37] Hawthorne B. S. Vella-Brodrick D. A. Hattie J. (2019). Well-being as a cognitive load reducing agent: A review of the literature. *Front. Educ.* 4:121. 10.3389/feduc.2019.00121

[B38] Huang X. Li S. Wang T. Lajoie S. P. (2024). The effects of emotion regulation and students’ perceived challenges on emotion synchrony in collaborative learning. *Res. Square*. 10.21203/rs.3.rs-3835295/v1 36284789

[B39] Kalyuga S. (2011). Cognitive load theory: How many types of load does it really need? *Educ. Psychol. Rev.* 23 1–19. 10.1007/s10648-010-9150-7

[B40] Kang S. H. (2016). Spaced repetition promotes efficient and effective learning: policy implications for instruction. *Policy Insights Behav. Brain Sci.* 3, 12–19. 10.1177/237273221562470

[B44] Kirschner P. Sweller J. Clark R. E. (2006). Why unguided learning does not work: An analysis of the failure of discovery learning, problem-based learning, experiential learning and inquiry-based learning. *Educ. Psychol.* 41 75–86. 10.1207/s15326985ep4102_1 42149202

[B45] Koole S. L. (2009). “Does emotion regulation help or hurt self-regulation,” in *Psychology of Self-Regulation: Cognitive, Affective, and Motivational Processes*, eds Forgas J. P. Baumeister R. F. Tice D. M. (Hove: Psychology Press), 217–231.

[B46] Koole S. L. (2010). The psychology of emotion regulation: An integrative review. *Cogn. Emot.* 23 4–41. 10.1080/02699930802619031

[B47] Koole S. L. Kuhl J. (2007). “Dealing with unwanted feelings: The role of affect regulation in volitional action control,” in *Handbook of Motivation Science*, eds Shah J. Gardner W. (New York, NY: Guilford Press).

[B48] Kosovich J. J. Hulleman C. S. Barron K. E. Getty S. (2015). A practical measure of student motivation: Establishing validity evidence for the expectancy-value-cost scale in middle school. *J. Early Adolescence* 35 790–816. 10.1177/0272431614556890

[B49] Krieglstein F. Beege M. Rey G. D. Sanchez-Stockhammer C. Schneider S. (2023). Development and validation of a theory-based questionnaire to measure different types of cognitive load. *Educ. Psychol. Rev.* 35:9. 10.1007/s10648-023-09738-0

[B52] Lim S. Y. Chapman E. (2013). Development of a short form of the attitudes toward mathematics inventory. *Educ. Stud. Math.* 82 145–164. 10.1007/s10649-012-9414-x

[B53] Mandler G. (2014). “Organization and repetition: Organizational principles with special reference to rote learning,” in *Perspectives on Memory Research (PLE: Memory)*, (Psychology Press), 293–327.

[B54] Marroquín B. (2011). Interpersonal emotion regulation as a mechanism of social support in depression. *Clin. Psychol. Rev*. 31 1276–1290. 10.1016/j.cpr.2011.09.005 21983267

[B55] Mauss I. B. Bunge S. A. Gross J. J. (2007). Automatic emotion regulation. *Soc. Pers. Psychol. Compass* 1 146–167. 10.1111/j.1751-9004.2007.00005.x

[B56] McGraw Hill, (2025). Available online at: https://www.mheducation.com/highered/digital-products/aleksppl (accessed January 5, 2025).

[B57] McRae K. (2016). Cognitive emotion regulation: A review of theory and scientific findings. *Curr. Opin. Behav. Sci.* 10 119–124. 10.1016/j.cobeha.2016.06.004

[B58] McVee M. B. Dunsmore K. Gavelek J. R. (2005). Schema theory revisited. *Rev. Educ. Res.* 75 531–566. 10.3102/00346543075004531

[B59] Meylani R. (2024). Innovations with schema theory: Modern implications for learning, memory, and academic achievement. *Int. J. Multidiscip. Res.* 6 2582–2160.

[B60] Middleton M. J. Midgley C. (2002). Beyond motivation: Middle school students’ perceptions of press for understanding in math. *Contemp. Educ. Psychol.* 27 373–391. 10.1006/ceps.2001.1101

[B61] Miyake A. Shah P. (1999). *Models of Working Memory.* Cambridge: Cambridge University Press, 442–481.

[B62] Moos D. Azevedo R. (2008). Self-regulated learning with hypermedia: The role of prior domain knowledge. *Contemp. Educ. Psychol.* 33 270–298. 10.1016/j.cedpsych.2008.02.001

[B66] Ochsner K. N. Gross J. J. (2005). The cognitive control of emotion. *Trends Cogn. Sci.* 9, 242–249. 10.1016/j.tics.2005.03.010 15866151

[B67] Ochsner K. N. Gross J. J. (2008). Cognitive emotion regulation: Insights from social cognitive and affective neuroscience. *Curr. Dir. Psychol. Sci.* 17 153–158. 10.1111/j.1467-8721.2008.00566.x 25425765 PMC4241349

[B68] Paas F. Renkl A. Sweller J. (2004). Cognitive load theory: Instructional implications of the interaction between information structures and cognitive architecture. *Instr. Sci.* 32 1–8. 10.1023/B:TRUC.0000021806.17516.d0

[B69] Paas F. Renkl A. Sweller J. (2003). Cognitive load theory and instructional design: recent developments. *Educ. Psychol.* 38, 1–4. 10.1207/S15326985EP3801_1 42149202

[B70] Pekrun R. (2006). The control-value theory of achievement emotions: Assumptions, corollaries, and implications for educational research and practice. *Educ. Psychol. Rev.* 18 315–341. 10.1007/s10648-006-9029-9

[B71] Pekrun R. Linnenbrink-Garcia L. (2022). “Academic emotions and student engagement,” in *The Handbook of Research on Student Engagement*, 2nd Edn, eds Reschly A. L. Christenson S. L. (Berlin: Springer), 109–132.

[B72] Pekrun R. Perry R. P. (2014). “Control-value theory of achievement emotions,” in *International Handbook of Emotions in Education*, eds Pekrun R. Linnenbrink-Garcia L. (New York, NY: Routledge), 120–141.

[B73] Pham M. T. (2007). Emotion and rationality: A critical review and interpretation of empirical evidence. *Rev. Gen. Psychol.* 11 155–178. 10.1037/1089-2680.11.2.155

[B74] Plass J. L. Kalyuga S. (2019). Four ways of considering emotion in cognitive load theory. *Educ. Psychol. Rev.* 31 339–359. 10.1007/s10648-019-09473-5

[B75] Plass J. L. Kaplan U. (2016). “Emotional design in digital media for learning,” in *Emotions, Technology, Design, and Learning*, eds Tettegah S. Y. Gartmeier M. (Cambridge, MA: Academic Press), 131–161.

[B76] Prinz J. (2012). “Emotions: How many are there?,” in *The Oxford Handbook of Philosophy of Cognitive Science*, eds Margolis E. Samuels R. Stich S. P. (Oxford: Oxford University Press). 10.1093/oxfordhb/9780195309799.013.0008

[B77] Russell J. A. (2003). Core affect and the psychological construction of emotion. *Psychol. Rev.* 110 145–172. 10.1037/0033-295X.110.1.145 12529060

[B78] Salomon G. (1984). Television is” easy” and print is” tough”: The differential investment of mental effort in learning as a function of perceptions and attributions. *J. Educ. Psychol.* 76 647–658. 10.1037/0022-0663.76.4.647

[B79] Scherer K. R. Moors A. (2019). The emotion process: Event appraisal and component differentiation. *Annu. Rev. Psychol.* 70 719–745. 10.1146/annurev-psych-122216-011854 30110576

[B81] Schwonke R. (2015). Metacognitive load – Useful, or extraneous concept? Metacognitive and self-regulatory demands in computer-based learning. *J. Educ. Technol. Soc.* 18 172–184.

[B82] Seufert T. (2018). The interplay between self-regulation in learning and cognitive load. *Educ. Res. Rev.* 24 116–129. 10.1016/j.edurev.2018.03.004

[B83] Seufert T. Hamm V. Vogt A. Riemer V. (2024). The interplay of cognitive load, learners’ resources and self-regulation. *Educ. Psychol. Rev.* 36:50. 10.1007/s10648-024-09890-1

[B85] Siemer M. (2001). Mood-specific effects on appraisal and emotion judgements. *Cogn. Emot.* 15 453–485. 10.1080/0269993004200178

[B86] Sweller J. (1988). Cognitive load during problem solving: Effects on learning. *Cogn. Sci.* 12 257–285. 10.1016/0364-0213(88)90023-7

[B87] Sweller J. (2010). Element interactivity and intrinsic, extraneous, and germane cognitive load. *Educ. Psychol. Rev.* 22 123–138. 10.1007/s10648-010-9128-5

[B88] Sweller J. (2011). “Cognitive load theory,” in *The Psychology of Learning and Motivation: Cognition in Education*, eds Mestre J. P. Ross B. H. (New York, NY: Elsevier Academic Press), 37–76. 10.1016/B978-0-12-387691-1.00002-8

[B89] Sweller J. van Merriënboer J. G. Paas F. (1998). Cognitive architecture and instructional design. *Educ. Psychol. Rev.* 10 251–296. 10.1023/A:1022193728205

[B90] Sweller J. van Merriënboer J. J. Paas F. (2019). Cognitive architecture and instructional design: 20 years later. *Educ. Psychol. Rev.* 31 261–292. 10.1007/s10648-019-09465-5

[B92] Turner J. C. Meyer D. K. (2004). A classroom perspective on the principle of moderate challenge in mathematics. *J. Educ. Res.* 97 311–318. 10.3200/JOER.97.6.311-318

[B93] Van Gog T. Kester L. Paas F. (2011). Effects of concurrent monitoring on cognitive load and performance as a function of task complexity. *Appl. Cogn. Psychol.* 25 584–587. 10.1002/acp.1726

[B94] van Merriënboer J. J. Sweller J. (2005). Cognitive load theory and complex learning: Recent developments and future directions. *Educ. Psychol. Rev.* 17 147–177. 10.1007/s10648-005-3951-0

[B97] Willner C. J. Hoffmann J. D. Bailey C. S. Harrison A. P. Garcia B. Ng Z. J. et al. (2022). The development of cognitive reappraisal from early childhood through adolescence: A systematic review and methodological recommendations. *Front. Psychol.* 13:875964. 10.3389/fpsyg.2022.875964 35814075 PMC9258621

[B98] Yang H. Yang S. Isen A. M. (2013). Positive affect improves working memory: Implications for controlled cognitive processing. *Cogn. Emot.* 27 474–482. 10.1080/02699931.2012.713325 22917664

